# Neuregulin-1 inhibits neuroinflammatory responses in a rat model of organophosphate-nerve agent-induced delayed neuronal injury

**DOI:** 10.1186/s12974-015-0283-y

**Published:** 2015-04-02

**Authors:** Yonggang Li, Pamela J Lein, Gregory D Ford, Cuimei Liu, Kyndra C Stovall, Todd E White, Donald A Bruun, Teclemichael Tewolde, Alicia S Gates, Timothy J Distel, Monique C Surles-Zeigler, Byron D Ford

**Affiliations:** Department of Neurobiology, Neuroscience Institute, Morehouse School of Medicine, 720 Westview Drive, SW, Atlanta, GA 30310 USA; Department of Molecular Biosciences, School of Veterinary Medicine, University of California, 1089 Veterinary Medicine Drive, Davis, CA 95616 USA; Department of Biology, Morehouse College, 830 Westview Drive SW, Atlanta, GA 30310 USA; Institute of Infectious Disease, Xiangya Hospital, Central-South University, No.9 Chegongzhuang Avenue, Changsha, 100044 China; Department of Physiology, Emory University, 201 Dowman Dr., Atlanta, GA 30322 USA

**Keywords:** Apoptosis, Chemokine, Cytokine, Delayed neurotoxicity, Immunity, Microarray, Neuroprotection, Nerve agent, Rat model

## Abstract

**Background:**

Neuregulin-1 (NRG-1) has been shown to act as a neuroprotectant in animal models of nerve agent intoxication and other acute brain injuries. We recently demonstrated that NRG-1 blocked delayed neuronal death in rats intoxicated with the organophosphate (OP) neurotoxin diisopropylflurophosphate (DFP). It has been proposed that inflammatory mediators are involved in the pathogenesis of OP neurotoxin-mediated brain damage.

**Methods:**

We examined the influence of NRG-1 on inflammatory responses in the rat brain following DFP intoxication. Microglial activation was determined by immunohistchemistry using anti-CD11b and anti-ED1 antibodies. Gene expression profiling was performed with brain tissues using Affymetrix gene arrays and analyzed using the Ingenuity Pathway Analysis software. Cytokine mRNA levels following DFP and NRG-1 treatment was validated by real-time reverse transcription polymerase chain reaction (RT-PCR).

**Results:**

DFP administration resulted in microglial activation in multiple brain regions, and this response was suppressed by treatment with NRG-1. Using microarray gene expression profiling, we observed that DFP increased mRNA levels of approximately 1,300 genes in the hippocampus 24 h after administration. NRG-1 treatment suppressed by 50% or more a small fraction of DFP-induced genes, which were primarily associated with inflammatory responses. Real-time RT-PCR confirmed that the mRNAs for pro-inflammatory cytokines interleukin-1β (IL-1β) and interleukin-6 (IL-6) were significantly increased following DFP exposure and that NRG-1 significantly attenuated this transcriptional response. In contrast, tumor necrosis factor α (TNFα) transcript levels were unchanged in both DFP and DFP + NRG-1 treated brains relative to controls.

**Conclusion:**

Neuroprotection by NRG-1 against OP neurotoxicity is associated with the suppression of pro-inflammatory responses in brain microglia. These findings provide new insight regarding the molecular mechanisms involved in the neuroprotective role of NRG-1 in acute brain injuries.

## Introduction

Organophosphorus (OP) nerve agents are rapidly acting and toxic chemicals that have been used by terrorists in military combat and against civilian populations [1,2]. A recent United Nations report confirmed that Syria used sarin gas in an attack against a civilian population [3], killing 1,400 people, including more than 400 children in the suburbs of Damascus. OP nerve agents were also used in Iraq against Kurdish civilians during the Iran-Iraq Gulf War of 1981 to 1987 [4]. In 1995, a Japanese doomsday cult used the nerve agent sarin to kill seven people and poison 600 others in an attack in the Japanese city of Matsumoto [5]. A year later, the cult used sarin in a terrorist attack on the Tokyo subway system that killed twelve and sent more than 5,000 people to hospitals [6-8]. As threats of terrorism increase, the development of therapeutic strategies for protecting the brain against the neurotoxic effects of OP nerve agents has become an important area of research.

OP nerve agents affect cholinergic neurotransmission by inhibiting the enzyme acetylcholinesterase (AChE). Current post-exposure medical countermeasures against nerve agents (for example, atropine, oximes, and benzodiazepines) are useful in preventing mortality but are not sufficiently effective in protecting the CNS from seizures and permanent injury [1]. We recently demonstrated the potential therapeutic benefit of neuregulin-1 (NRG-1) in a rat model of acute OP poisoning [9]. NRG-1 belongs to a family of multipotent neuroprotective and anti-inflammatory growth factors that include acetylcholine receptor inducing activities (ARIAs), glial growth factors (GGFs), heregulins, and neu differentiation factors (NDFs) [10-14]. Our studies showed that NRG-1 reduced delayed neuronal death by approximately 90% in multiple brain regions of rats acutely intoxicated with the OP diisopropylfluorophosphate (DFP) when administered up to 1 h following DFP intoxication [9].

There is strong evidence that inflammatory reactions are involved in OP-mediated neuronal injury and result in poor prognosis of neurological outcome [2,15-19]. Brain microglial cells are rapidly activated in response to OP nerve agents [20,21], and inflammatory cytokines, such as interleukin-1 (interleukin-1α (IL-1α) and interleukin-1β (IL-1β)), interleukin-6 (IL-6), and tumor necrosis factor α (TNFα) are induced in microglia and other cells in the rodent brain following OP intoxication [22-27].

Therefore, in this study, we examined whether NRG-1 prevents OP-induced pro-inflammatory responses in the brain. Our findings indicate that NRG-1 suppressed DFP-induced microglial activation and brain levels of mRNA encoding the pro-inflammatory cytokines IL-1β and IL-6. These results may yield insight into the mechanisms involved in the neuroprotective efficacy of NRG-1 in nerve-agent-induced brain injury.

## Methods

### Animals and DFP exposures

All animals used in these studies were treated humanely and with regard to alleviation of suffering and pain, and all protocols involving animals were approved by the Institutional Animal Care and Use Committee (IACUC) of Morehouse School of Medicine, Oregon Health & Science University and University of California, Davis (UCD) prior to the initiation of experimentation. Adult male Sprague–Dawley rats (280 to 320 g; Harlan Laboratories, USA) were housed in standard plastic cages in a temperature-controlled room (22°C ± 2°C) on a 12-h reverse light–dark cycle. Food and water were provided *ad libitum*. Animals were anesthetized with 2% isoflurane (30% oxygen, 70% nitrous oxide) and injected i.m. with pyridostigmine bromide (PB; P1339, TCI America, Portland, OR) at 0.1 mg/kg body weight (BW) in saline and with atropine methylnitrate (AMN; A0755, TCI America) at 20 mg/kg BW in saline 30 and 10 min prior to DFP injection, respectively. AMN and PB do not readily cross the blood brain barrier, so these drugs are centrally inactive [28] but effectively block peripheral OP neurotoxicity, thereby reducing mortality and facilitating detection of seizure symptoms [29,30]. Animals were then injected i.p. with DFP (D0879, Sigma Chemical Co., St. Louis, MO) at 9 mg/kg BW diluted in sterile distilled water as previously described [9,30]. DFP was always prepared fresh within 5 min before administration. The intensity of seizures in the delayed neuronal injury model was evaluated using a 5-point ranking system specifically designed to measure seizure activity in animals pre-treated with peripheral antidotes [29,31]. In paradigm, seizure activity is noted only during the first hour post-DFP exposure, and we observed that animals that do not have tonic-clonic seizures during that hour also do not exhibit injured neurons [30]. The animals were observed for a period of 1 h after drug administrations and animals not showing seizure activity were excluded. AChE activity was determined as previously described [30].

### Intra-arterial administration of NRG-1

The left common carotid artery (CCA) was exposed in anesthetized animals through a midline incision and was carefully dissected free from surrounding nerves and fascia [32]. The occipital artery and superior thyroid branches of the external carotid artery (ECA) were isolated and electrocoagulated. The ECA was dissected further distally. The internal carotid artery (ICA) was isolated and carefully separated from the adjacent vagus nerve, and the pterygopalatine artery was ligated close to its origin with a 6–0 silk suture. Animals were randomized, and NRG-1 or vehicle was administered via the ECA as a 10-μl single bolus of NRG-1β EGF-like domain (R&D Systems, Minneapolis, Minnesota) at 3.2 μg/kg (in phosphocitrate buffer, pH 5.0 with 1% BSA) using a Hamilton syringe. The dose of NRG-1 was selected based on previous studies of the neuroprotective efficacy of NRG-1 against DFP neurotoxicity [9]. All surgical procedures were performed using sterile/aseptic techniques in accordance with IACUC guidelines. Animals were selected and randomized before DFP intoxication and treated with either NRG-1 (*n* = 7) or vehicle (phosphocitrate buffer, pH 5.0 with 1% BSA; *n* = 7). NRG-1 and vehicle were administered inter-arterially 5 min prior to DFP injection. In all studies, anesthesia was stopped immediately following injection of DFP. Rectal temperature was maintained between 36.5°C and 37.0°C during anesthesia with a Homeothermic Blanket Control Unit (Harvard Apparatus, Holliston, MA).

### Histology and immunohistochemistry

At 24 h post-DFP injection, rats were deeply anesthetized with 5% isoflurane and perfused transcardially with saline followed by cold 4% PFA solution in PBS for 30 min. Brains were quickly removed and cryoprotected in 30% sucrose. Coronal sections of 20 μm thickness were cryosectioned from the entire brain of each animal. Sections were mounted on slides which were stored at −80°C until further processed. Fluro-Jade B (FJB, AG310, Millipore, Billerica, MA) labeling was performed as previously described [30]. For immunostaining, after rinsing in 0.01 M PBS, sections were blocked with PBS containing 5% normal goat serum and 0.1% triton X-100 for 1 h at 4°C. Sections were then incubated for 1 h at 37°C with mouse monoclonal anti-ED1 (1:500, MAB1435, Millipore) and anti-CD11b (1:500, CBL1512, Millipore) antibodies. Sections were washed with PBS and incubated with a Cy3-conjugated goat anti-mouse IgG antibody (1:400, 115-165-166, Jackson Laboratories, Bar Harbor, ME) for 1 h at room temperature. All negative controls were incubated with PBS instead of the primary antibodies. For dual labeling studies, sections were first processed for immunohistochemistry as described above, and then processed using a modified method for FJB labeling. The modified protocol included brief immersion in 0.015% KMnO4 for 1 min followed by incubation in a 0.0001% solution of FJB solution for 8 min. These modifications reduced the loss of immunohistochemical staining and minimized fluorescent bleed-through of FJB labeling during microscopy. The sections were rinsed with distilled water and cover slipped with mounting medium containing 0.1% acetic acid and 80% glycerin. Fluorescence was visualized via indirect fluorescence microscopy.

### RNA isolation and real-time reverse transcription polymerase chain reaction

Total RNA was isolated from the hippocampus, cortex, and the entire subcortex of each brain using TRIzol reagent (Life Technologies, Grand Island, NY; *n* = 7 to 9, per condition). RNA was treated with DNase I to remove any traces of genomic DNA. First-strand cDNA was synthesized from 1 μg of each RNA sample using oligo (dT) and Omniscript reverse transcriptase (Qiagen, Valencia, CA, USA) according to the manufacturer’s protocol. Each set of samples was simultaneously processed for RNA extraction, DNase I treatment, cDNA synthesis, and PCR reaction. Real-time reverse transcription polymerase chain reaction (RT-PCR) was performed with SYBR Green (IQTM Sybr Green Supermix, BioRad, Hercules, CA, USA) using an iCycler (BioRad, Hercules, CA, USA). To quantify mRNA expression, primers for rat IL-1β (sense, 5′-aggcttccttgtgcaagtgt-3′; antisense, 5′-tgagtgacactgccttcctg-3′), IL-6 (5′-ccggagaggagacttcacag-3′; antisense, 5′-cagaattgccattgcacaac-3′), TNFα (sense, 5′-agatgtggaactggcagagg-3′, antisense, 5′-cccatttgggaacttctcct-3′), and glyceraldehyde-3-phosphate dehydrogenase (GAPDH; sense, 5′-acccagaagactgtggatgg-3′; antisense, 5′-cacattgggggtaggaacac-3′) were used. Cycling parameters were 95°C 3.5 min, then 40 cycles of 95°C for 10 s, 55°C for 45 s, 95°C 1 min, 55°C 1 min, and then melt following the manufacturer’s protocol. The fluorescence of the accumulating product was measured at the product melting temperature. To confirm the specificity of PCR products, melting curves were determined using iCycler software. mRNA levels were normalized against GAPDH mRNA levels in the same sample. GAPDH mRNA levels did not show any significant treatment-related variation in our experiment. PCR results are from naive animals and control (PB + AMN + water), DFP (PB + AMN + DFP + vehicle), and DFP + NRG-1 (PB + AMN + DFP + NRG-1)-treated animals. Intensity values are means ± SEM. Statistical analysis was carried out using ANOVA. Differences were considered significant at the level of *P* < 0.05.

### RNA preparation and GeneChip microarray analysis

Microarray analysis was performed using Affymetrix GeneChips (Affymetrix, Santa Clara, CA, USA). Animals were sacrificed 24 h after DFP and NRG-1 administration, and hippocampal tissues from control, DFP treated, DFP + NRG-1 treated animals (*n* = 3 per condition) were used for subsequent RNA isolation. Total RNA was extracted with TRIzol Reagent (Life Technologies, Grand Island, NY, USA) followed by a further cleanup with the Ambion RNAqueous kit (RNAqueous Kit, Ambion/Life Technologies, Grand Island, NY). An Agilent bioanaylzer was used to measure RNA integrity and the NanoDrop to measure RNA quantity. cRNA was synthesized using a GeneChip 3′ IVT Express Kit according to the manufacturer’s protocol (Affymetrix, Santa Clara, CA, USA). One hundred nanograms of RNA was used for the microarrays. Total RNA was reversed transcribed to synthesize first-strand cDNA and then converted into a double-stranded DNA. Amplified RNA (aRNA) was synthesized by *in vitro* transcription and labeled by incorporating a biotin-conjugated nucleotide into the molecule. The aRNA was then purified and fragmented for hybridization onto GeneChip 3′ expression arrays. Biotinylated aRNA was hybridized to an Affymetrix Rat Genome U230 2.0 GeneChip with approximately 30,000 transcripts. The chips were hybridized at 45°C for 16 h, and then washed, stained with streptavidin-phycoerythrin, and scanned according to manufacturing guidelines.

### Affymetrix microarray data analysis

We used this dataset to further examine the transcriptional regulation of genes induced by DFP and suppressed by NRG-1. Initial data analysis was performed using Affymetrix Expression Console software (Affymetrix, Santa Clara, CA, USA). Affymetrix microarrays contain the hybridization, labeling, and housekeeping controls to evaluate the success of the hybridizations. Affymetrix Transcriptome Analysis Console (TAC) Software performed statistical analysis to enable the identification of differentially expressed genes. Gene expression values that increased by twofold or more in TAC were determined statistically significant (*P* < 0.005) using one-way ANOVA and a threshold of the false discovery rate (FDR) based on the Benjamini-Hochberg step-up FDR-controlling procedure. Three chips were used for each experimental group: control, DFP, and DFP + NRG-1. Genes in the hippocampus of DFP intoxicated animals that increased in expression by twofold or more compared to control and were decreased twofold or more by NRG-1 were identified and further analyzed. Principal component analysis (PCA) was conducted using the Gene Expression Similarity Investigation Suite software (Genesis; http://genome.tugraz.at/genesisclient/genesisclient_description.shtml). Genesis uses a three-dimensional coordinate system, where the x-axis represents the principal component 1 (PC1), the y-axis PC2, and the z-axis PC3. Data points near to each other in the PC space are similar in gene expression, whereas data points that are far apart from each other in the three-dimensional space are not similar to each other in gene expression.

### Ingenuity Pathway Analysis

The DFP-induced genes that were attenuated by NRG-1 were analyzed using Ingenuity Pathway Analysis (Ingenuity® Systems; http://www.ingenuity.com/products/ipa) and overlaid onto a global molecular network developed from information contained in the Ingenuity Pathways Knowledge Base. Fischer’s exact test was used to calculate a *P* value determining the probability that each biological function and/or canonical pathway or gene network identified is due to change alone. The canonical pathways that were most statistically relevant to the dataset were identified. We overlaid the gene expression profiles on the canonical pathway and gene network figures to reveal similarities and dissimilarities in their gene expression patterns.

## Results and discussion

### Neuregulin-1 inhibits DFP-induced microglial activation

**Figure 1:**
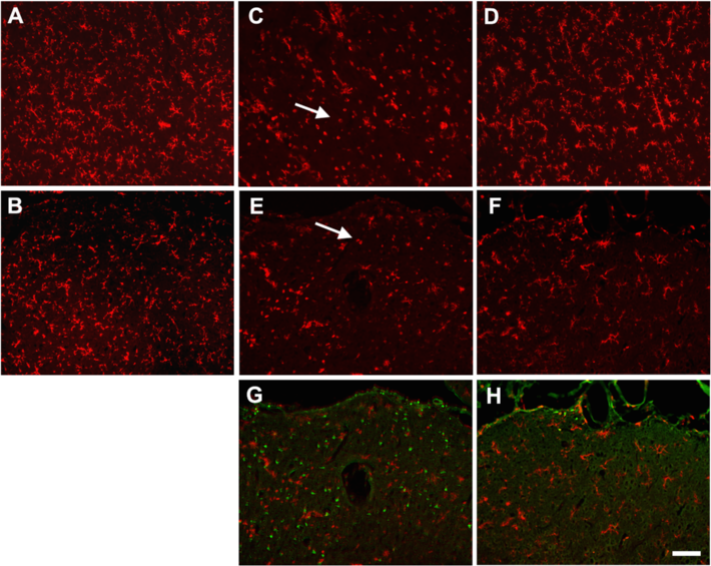
**Neuregulin-1 inhibits DFP-induced changes in microglial morphology in the cortex and thalamus.** Anesthetized rats were pretreated with PB (0.1 mg/kg, i.m.) and AMN (20 mg/kg, i.m.) 30 and 10 min, respectively, prior to i.p. injection of DFP (9 mg/kg). NRG-1 or vehicle was administered via injection into the carotid artery 5 min prior to DFP exposure. Control animals received PB, AMN, and water in place of DFP. The CD11b antibody was used to identify resting and activated microglia in superficial layers of cortex **(A, C, D)** and in the lateral dorsal thalamus **(B, E, F, G, H)** 24 h following DFP administration. CD11b immunopositive cells (red labeling) in the cortex **(A)** and thalamus **(B)** of control animals displayed the characteristic ramified morphology of resting microglia. In contrast, CD11b immunopositive cells in these same brain regions of DFP-intoxicated animals exhibited morphology characteristic of activated microglia (indicated by arrows), for example, increased size of the cell body, thickened proximal processes, and decreased ramification of distal branches **(C, E, G)**. CD11b immunopositive cells in DFP intoxicated animals treated with NRG-1 **(D, F, H)** exhibited morphologies that more closely resembled those observed in controls. Dual labeling with CD11b and FJB (green labeling) showed that in the thalamus, activated microglia were present in areas with significant neurodegeneration following DFP **(G)**, but neither activated microglia nor injured neurons were present in the thalamus of DFP intoxicated animals pretreated with NRG-1 **(H)**. Scale bar = 50 μm.

**Figure 2:**
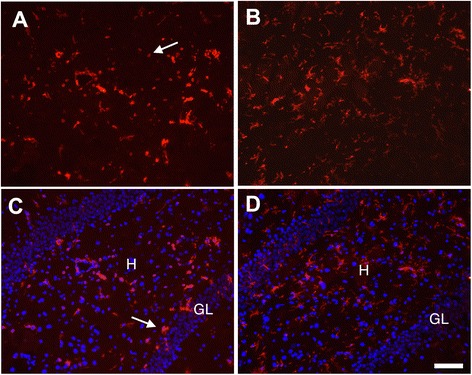
**Neuregulin-1 inhibits DFP-induced changes in microglial morphology in the hippocampus.** Brains collected 24 h post-DFP administration were immunostained for CD11b to identify microglia in the hippocampus, and a subset of these sections was also stained for DAPI **(C, D)**. CD11b immunopositive cells with morphological characteristics of activated microglia (indicated by arrows) were seen in the hippocampus 24 h following DFP administration **(A, C)**. In contrast, CD11b immunopositive cells in the hippocampus of DFP intoxicated animals treated with NRG-1 exhibited morphologies characteristic of resting microglia **(B, D)**. H, hilus; GL, granule cell layer. Stereotaxic coordinate = bregma −3; Scale bar = 50 μm.

DFP is structurally and toxicologically similar to the OP nerve agents and thus is used as an OP nerve agent stimulant in experimental animal models [29,30]. We previously demonstrated that rats injected with DFP at 9 mg/kg, i.p., experience seizures and exhibited significant delayed neurodegeneration in multiple brain regions [9]. Microglial activation is a characteristic brain inflammatory response induced following OP nerve agent intoxication [20,21]. Under normal physiological conditions, resting microglia display a ramified state; however, when activated, microglia undergo a morphological transformation from the resting ramified state to an amoeboid shape. To determine the effects of acute DFP intoxication on microglia, brain sections from rats injected with vehicle or DFP in the absence or presence of NRG-1 were immunostained for CD11b, a biomarker of microglia [33]. Microglia in the superficial layers of cortex (Figure 1A) and lateral dorsal thalamus (Figure 1B) in control animals displayed the characteristic ramified morphology of resting microglia. Acute intoxication with DFP caused microglial activation, as indicated by the increased size of the cell body, a thickening of proximal processes, decreased ramification of distal branches and/or amoeboid shaped cell bodies of CD11b immunopositive cells (Figure 1C, E). NRG-1 treatment prevented the DFP-induced morphological changes of microglial cells in those brain regions (Figure 1D, F) as CD11b immunopositive cells were morphologically similar to microglia in control brains. Dual labeling with CD11b and FJB showed that in the thalamus of animals acutely intoxicated with DFP, activated microglia were detected in areas of brain injury (Figure 1G). However, neither activated microglia nor injured neurons were present in the thalamus of DFP intoxicated animals treated with NRG-1 (Figure 1H). We previously showed that DFP administration resulted in neuronal injury in the CA1, CA3, and dentate gyrus of the hippocampus which was attenuated by NRG-1 [9]. Similarly, microglial activation was also seen in the hippocampus, including the dentate gyrus shown here, following DFP intoxication (Figure 2A, C). The microglial response to DFP was attenuated by NRG-1 treatment (Figure 2B, D). NRG-1 treatment did not prevent microglial activation in the amygdala, medial dorsal thalamus, reunion area of thalamus or piriform cortex of the DFP intoxicated animal (data not shown), brain regions in which neuronal injury was previously shown to not be protected by NRG-1 [9].

**Figure 3:**
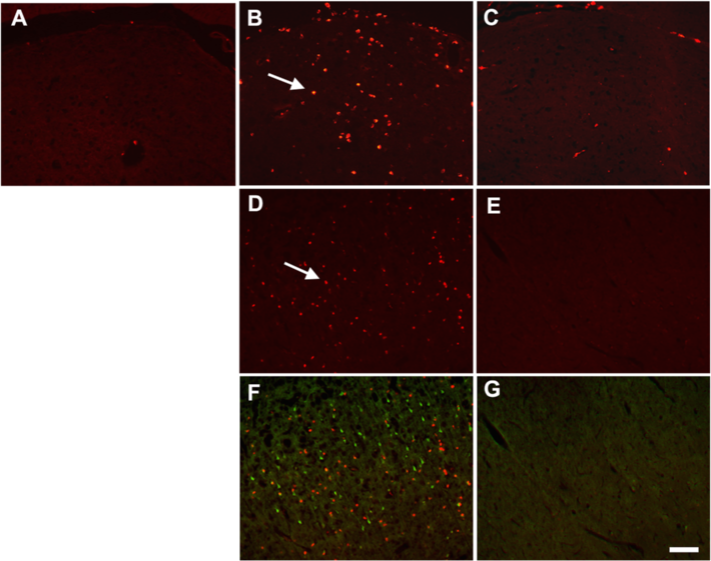
**Neuregulin-1 blocks DFP-induced microglia activation in the cortex and thalamus.** The ED-1 antibody was used to identify macrophages and activated microglia 24 h following DFP and NRG-1 administration. There was virtually no ED1 immunoreactivity detected (red labeling; arrows) in the brain of control animals **(A)**, but there were numerous ED1 immunopositive cells detected in the thalamus **(B)** and cortex **(D)** of DFP treated animals. Treatment with NRG-1 significantly decreased the number of ED1 immunopositive cells detected in the thalamus **(C)** and cortex **(E)** of DFP intoxicated animals. Dual labeling for ED-1 and FJB (green labeling) showed that activated microglia were seen in areas of the DFP-exposed cortex with significant neurodegeneration **(F)** and that neither activated microglia nor injured neurons were observed in the cortex of DFP-intoxicated animals that were pretreated with NRG-1 **(G)**. Scale bar = 50 μm.

**Figure 4:**
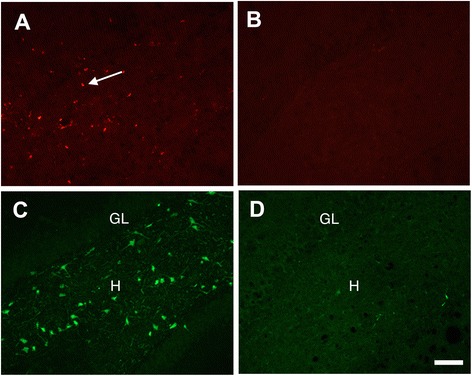
**Neuregulin-1 blocks DFP-induced microglia activation in the hippocampus.** Labeling of adjacent sections from the dentate gyrus of the hippocampus showed that in DFP intoxicated animals, ED-1 immunoreactivity (**A**; arrows) coincided with regions of FJB stained neurons **(C)**. In contrast, in DFP intoxicated animals pretreated with NRG-1, virtually no ED-1 immunoreactivity **(B)** or FJB staining **(D)** was observed. H, hilus; GL, granule cell layer. Stereotaxic coordinate = bregma −3; Scale bar = 50 μm.

To confirm the data based on morphological criteria of resting versus activated microglia, brain sections were also immunostained using the ED-1 antibody, which is a specific biomarker of macrophages and activated microglia [33]. There was virtually no ED1 immunoreactivity in the brains of vehicle control animals, including dorsal lateral thalamus (Figure 3A). However, there were numerous ED-1 immunopositive macrophages/activated microglia detected in brain regions of DFP-intoxicated animals. ED-1 immunopositive cells were amoeboid shaped (characteristic of activated macrophages and microglia) and distributed throughout the dorsal lateral thalamus (Figure 3B) and the superficial layers of cortex (Figure 3D). However, in DFP-intoxicated animals pre-treated with NRG-1, only a few ED-1 immunopositive cells were seen in injured regions of the dorsal lateral thalamus (Figure 3C) and no ED-1 immunopositive cells were observed in the cortex (Figure 3E). Dual labeling for ED-1 and FJB showed that activated microglia were associated with areas of neuronal injury in the cortex (Figure 3F) of DFP-intoxicated animals. Neither activated microglia nor injured neurons were observed in the cortex of DFP-intoxicated animals pre-treated with NRG-1 (Figure 3G). Similar to observations of CD11b immunolabeling, there was no change in the density of ED1 immunopositive cells in the amygdala, reunion area of thalamus, medial dorsal thalamus, and piriform cortex (data not shown), which were previously shown to be non-responsive to the neuroprotective effects of NRG-1 [9]. DFP induced ED-1 immunoreactivity in the hippocampus (Figure 4A), which was significantly reduced by NRG-1 pre-treatment (Figure 4B). Labeling of adjacent hippocampal sections of the dentate gyrus with ED-1 and FJB indicated that ED-1 immunoreactivity was detected both within and outside the hilar region where there were significant numbers of FJB stained neurons (Figure 4C) but in the hippocampi of DFP-intoxicated rats treated with NRG-1, there was no FJB labeling (Figure 4D).

### NRG-1 prevents DFP-induced hippocampal gene expression

**Figure 5:**
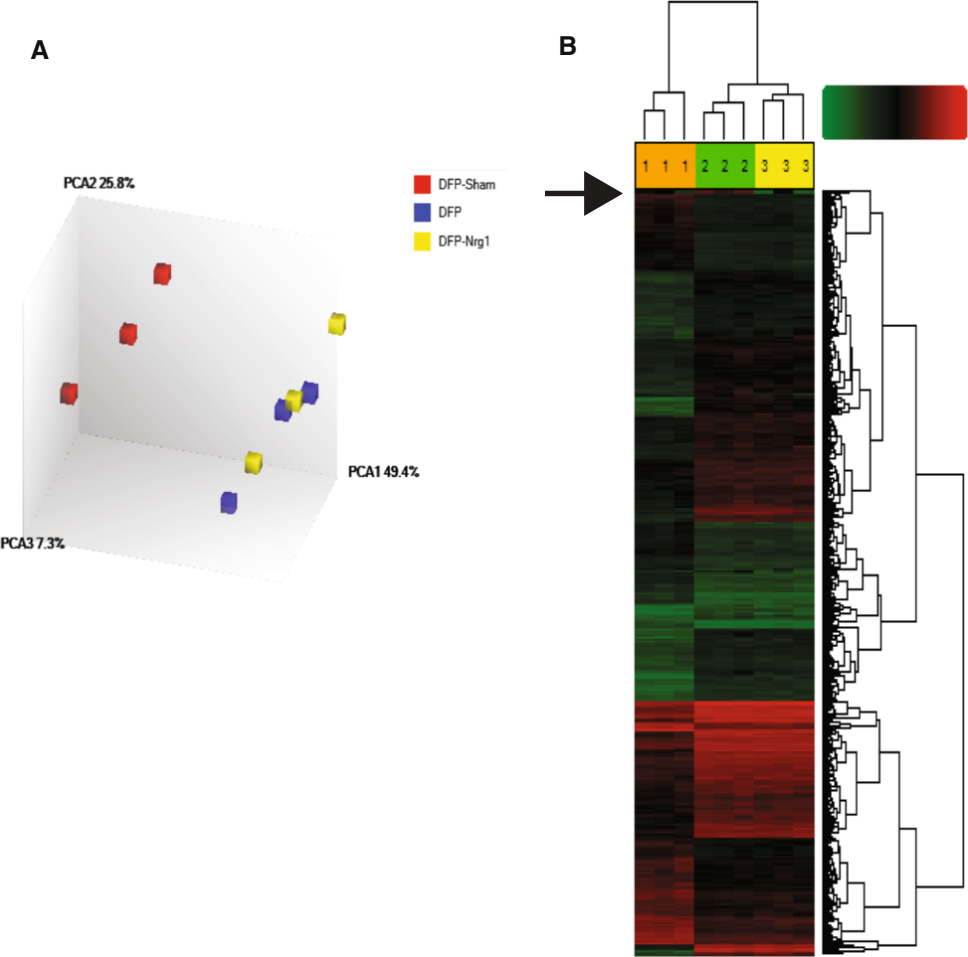
**NRG-1 prevents DFP-induced hippocampal gene expression.** Gene expression profiles in the hippocampus of three treatment groups (control, DFP, and DFP + NRG-1) were determined by mRNA microarray using the rat genome U230 2.0 GeneChip that contains approximately 30,000 probe sets (transcripts). **(A)** To assess inter-experiment differences in the intensity of gene expression, replicate experiments were compared for each experimental group and the resulting datasets analyzed by principle component analysis (PCA). The control samples clustered together and were distinct from and not overlapping with the DFP and DFP + NRG-1 groups, which were similar to each other. **(B)** Hierarchical cluster analysis graphically illustrates that approximately 1,300 genes were induced following DFP administration and that the induction of a small subset of these (indicated by the arrow) were attenuated by NRG-1 pretreatment. Green indicates genes with low level or no expression and red indicates genes with increased expression levels. Three animals were compared from each treatment group: (1) control; (2) DFP; and (3) DFP + NRG-1. DFP, diisopropylflurophosphate; NRG-1, neuregulin-1.

To examine gene expression profiles, total RNA was isolated from the hippocampus of each experimental group (control, DFP, and DFP + NRG-1) at 24 h post-DFP injection and interrogated using the rat genome U230 2.0 GeneChip that contains approximately 30,000 probe sets (transcripts). To assess inter-experiment differences in the intensity of gene expression, replicated experiments were compared for each experimental group and analyzed by principle component analysis (PCA). Similarity between data points in the PCA was determined by the distance between the points in the three-dimensional plot generated, with shorter distance indicating increased similarity (Figure 5A). Each data point represents one animal’s gene expression profile. Control, DFP, and DFP + NRG-1 gene expression profiles clustered within treatment groups, but the control group was well isolated from the other two treatment groups and the profiles for DFP and DFP + NRG-1 treatment groups were similar. To examine the changes in gene expression, we compared the gene expression profiles of the vehicle controls to the DFP animals and the gene expression profiles of the DFP animals to the DFP + NRG-1 animals. Figure 5B is a heat map illustrating that many genes were induced following DFP administration and that a very small subset of those genes were attenuated by NRG-1 treatment.

**Figure 6:**
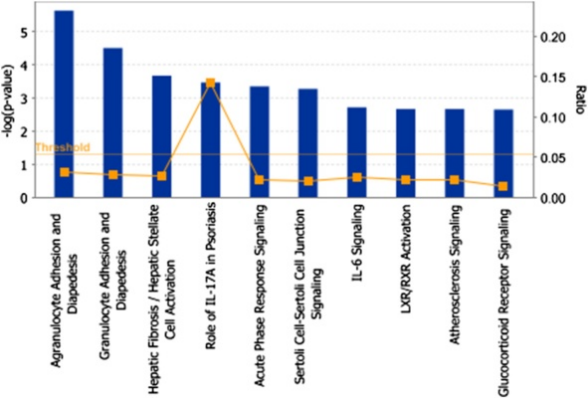
**Pathway analyses of gene differentially regulated by NRG-1 pretreatment of DFP intoxicated animals.** DFP-induced genes that were suppressed 50% or more by pretreatment with NRG-1 were analyzed using Ingenuity Pathway Analysis (IPA) software to identify canonical pathways associated with this subset of genes. IL, interleukin.

The set of genes induced by two-fold or more at 24 h following DFP administration (1,298 transcripts) and that were suppressed 50% or more by treatment with NRG-1 (41 transcripts) was imported into the IPA software to examine functions and pathways associated with this subset of genes. Analysis of the top ten canonical pathways associated with the dataset in IPA shows that most of these pathways are associated with immune function (Figure 6). Immune cell trafficking (agranulocytes and granulocyte adhesion and diapedesis), cytokine (IL-17 and IL-6) signaling, and acute phase response signaling were ranked among the most significant pathways, consistent with a key role for inflammation in mediating the delayed neural injury associated with OP neurotoxicity. The full list of genes that passed the fold change and false discovery rate cutoffs, including names and the GenBank accession numbers, is provided in Table 1. The DFP-induced genes that were significantly attenuated by NRG-1 treatment included the cytokine IL-1β, chemokines CXCL2 and CXCL11, and leukocyte cell derived chemotaxin-1 (LECT1). DFP increased the expression of paraoxonase-1 (PON1), an enzyme involved in the hydrolysis of organophosphates and NRG-1 suppressed the induction of PON-1 by DFP.

**Table 1:** **Genes induced 2-fold by DFP and suppressed 50% or more by NRG-1**

**Transcript cluster ID**	**Gene symbol**	**Description**	**FC OP vs. control**	**FC OP/NRG-1 vs. OP**
1367794_at	A2m	Alpha-2-macrolobulin	2.34	−2
1369625_at	Aqp1	Aquaporin 1	5.1	−17.93
1383946_at	Cldn 1	Claudin 1	3.94	−5.7
1375933_at	Cldn 2	Claudin 2	2.44	−25.97
1393891_at	Col8a 1	Collagen, type VIII, alpha 1	2.73	−5.42
1374172_at	Col8a 2	Collagen, type VIII, alpha 2	3.37	−19.49
1370391_at	Crabp 2	Cellular retinoic acid binding protien 2	2.18	−2.04
1379365_at	Cxcl 11	Chemokine (C-X-C motif) ligand 11	17.41	−2.13
1368760_at	Cxcl 2	Chemokine (C-X-C motif) ligand 2	2.67	−2.61
1367700_at	Fmod	Fibromudolin	2.02	−8.91
1387889_at	Folr 1	Folate receptor 1 (adult)	3.9	−12.25
1368337_at	Glycam	Glycosylation-dependent cell adhesion molecule 1	3.81	−7.01
1393840_at	Gnat 2	Guanine nucleotide protein (G protein), alpha transducing activity polypeptide 2	2.68	−2.52
1391915_at	Hspa 9	Heat shock protein 9	2.02	−2.13
1398256_at	II 1b	Interleukin 1 beta	3.08	−2.13
1387050_s_at	Kng 1	Kininogen 1; kininogen 1-like 1; kininogen 2	3.46	−3.31
1387868_at	Lbp	Lipopolysaccharide binding protien	3.31	−2.62
1387164_at	Lect 1	Leukocyte cell derived chemotaxin 1	4.8	−5.56
1393210_at	LOC100361383	Extracellular matrix protein 2-like	2.11	−2.09
1375465_at	Otx 2	Homeobox protein OTX2-like; orthodenticle homeobox 2	2.51	−17.47
1375908_at	Mpzl 2	Myelin protein zero-like 2	4.06	−4.16
1368302_at	Msx1	Msh homeobox 1	2.04	−5.63
1371849_at	Nt5dc2	5′-nucleotidase domain 2	2.13	−4.9
1385248_a_at	Ogn	Osteoglycin	2.38	−2.16
1375367_at	Pdlim	PDZ and LIM domain 2	2.67	−3.73
1370068_at	Pla2g5	Phospholipase A2, group V	3.01	−7.5
1371050_at	Pon1	Paraoxonase 1	2.48	−3.99
1370012_at	Ptgis	Prosta landin l2 (prostacyclin) synthase	2.75	−2.74
1380334_at	Rbm47	RNA binding motif protein 47	2.11	−3.46
1385500_at	RGD1561795	Similar to RIKEN cDNA 1700012B09	2.96	−5.52
1387125_at	S100a9	S100 calcium binding protein A9	6.6	−2.88
1377034_at	Serpin1a	Serine (or cysteine) proteinase inhibitor, clade B, member 1a	3.07	−2.2
1385005_at	Slc22a8	Solute carrier family 22 (organic anion transporter), member 8	2.12	−3.45
1368606_at	Slco1a5	Solute carrier organic transporter family, member 1a5	4.34	−48.37
1367998_at	Slpi	Secretory leukocyte peptidaase inhibitor	5.12	−2.32
1390525_a_at	Stra6	Stimulated by retinoic acid gene 6	2.64	−4.31
1384522_at	Sytl3	Synaptotagmin-like 3	2	−2.06
1383606_at	Tc2n	Tandem C2 domains, nuclear	4.01	−3.59
1387013_at	Tmem27	Transmembrane protein 27	4.53	−34.51
1388557_at	Tubb4b	Tubulin, beta 4B class IVb	2.24	−4.21

### NRG-1 reduces DFP-induced mRNA levels of pro-inflammatory cytokines in the brain

**Figure 7:**
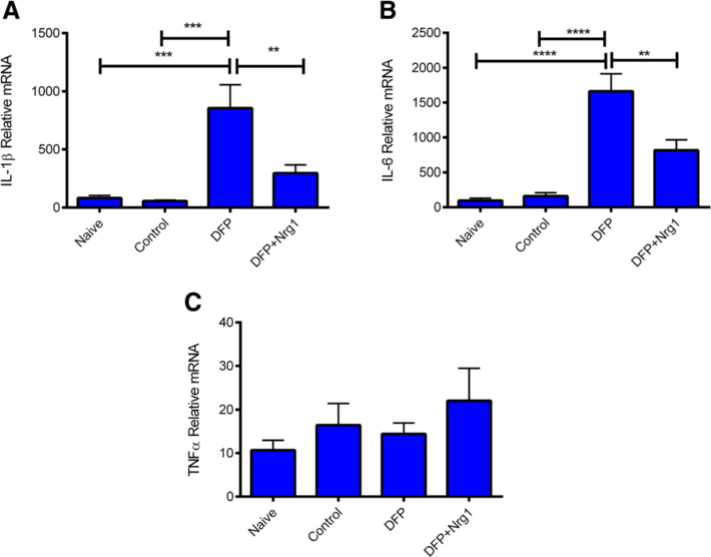
**NRG-1 reduces DFP-induced expression of pro-inflammatory cytokine mRNA in the brain.** RNA was isolated from the hippocampus and analyzed by RT-PCR to quantify the expression of mRNA for IL-1β **(A)**, IL-6 **(B)**, and TNFα **(C)** (*n* = 7 to 9 biological replicates for each condition). The data are presented as mean ± SEM of the relative mRNA (in arbitrary values) levels in naive, control, DFP, and DFP + NRG-1-treated rats after normalization to GAPDH mRNA levels (**P < 0.01; ***P < 0.001; ****P < 0.0001).

To confirm the microarray results indicating an effect of NRG-1 on pro-inflammatory cytokine mRNA expression, we used real-time RT-PCR to determine the levels of IL-1β, IL-6, and TNFα in hippocampal tissues 24 h following DFP and NRG-1 administration. Microarray results showed that IL-1β and IL-6, but not TNFα, were upregulated by DFP. Pretreatment with NRG-1 reduced levels of DFP-induced IL-1β mRNA by more than 50% of the levels observed in DFP intoxicated animals. DFP-induced levels of IL-6 mRNA were also reduced by NRG-1 but by less than the 50% cutoff, and NRG-1 treatment did not affect levels of DFP-induced TNFα mRNA. Similarly, real-time RT-PCR results showed that IL-1β and IL-6 were strongly induced by DFP in the brain while relatively little was seen in control brain tissues (Figure 7A, B). Consistent with the microarray results, DFP-induced IL-1β and IL-6 mRNA levels were reduced by NRG-1. TNFα levels were unchanged by either DFP or NRG-1 at 24 h post-DFP injection (Figure 7C).

Although there has been progress with efforts to treat patients with acute symptoms following OP exposure, strategies to prevent the subsequent OP-induced delayed neuronal injury are currently unavailable. It is well established that OP intoxication results in the stimulation of pro-inflammatory responses that lead to neuronal injury and increased neurological impairment [2,15-19]. OPs initiate inflammatory responses in the injured brain that progress for days after the onset of symptoms. Experimental studies show that inflammatory reactions in the brain can enhance neuronal excitability and impair cell survival, and conversely, some anti-inflammatory treatments reduce brain pathology in animal models of acute CNS injury [2,34]. Therefore, interventions that are aimed at decreasing neuroinflammation have potential as therapeutic agents for treating humans poisoned with OP neurotoxins and nerve agents.

Recent studies from our lab showed that NRG-1 treatment significantly reduced DFP-induced delayed neuronal damage and oxidative stress in rats when administered up to 1 h following DFP intoxication [9,30]. One of the early consequences of OP intoxication is the activation of brain microglia [2,20,21,35], therefore, in this study, we examined whether NRG-1 could regulate DFP-induced microglial activation in the brain. Our data demonstrated that NRG-1 blocked DFP-induced morphological changes in microglia associated with activation and subsequent delayed neuronal damage. The region specific anti-inflammatory effects of NRG-1 are consistent with our findings showing selective DFP-induced neuronal injury and neuroprotection by NRG-1 [9,30]. We proposed that the effects could be the result to several factors, including access of exogenously administered NRG-1 to various brain regions, selective vulnerability of specific neurons and/or and regional differences in NRG-1 receptor expression.

To elucidate the molecular mechanisms used by NRG-1 to block microglia activation and neuronal injury, we examined gene expression profiles in hippocampal brain tissues following DFP intoxication and NRG-1 treatment. DFP increased the expression of nearly 1,300 transcripts by twofold or more, however only 41 of those genes were suppressed 50% or more by NRG-1. Bioinformatic analysis of the NRG-1 suppressed genes with IPA software indicated that immune cell trafficking, hepatic fibrosis/hepatic stellate cell activation, acute phase response, and IL-6 signaling were among the top canonical pathways associated with the anti-inflammatory and neuroprotective effects of NRG-1. These findings are similar to studies that analyzed gene expression profiles of the prefrontal cortex from rats 24 h after soman exposure [36].

Activated brain microglia release pro-inflammatory cytokines following OP poisoning, which are toxic to neurons [2,22-27]. Inflammatory cytokines, such as IL-1α, IL-1β, IL-6, and TNFα are induced in the rodent brain following OP intoxication [22-27]. IL-1β is primarily expressed by activated brain microglia following OP exposure [25]. NRG-1 attenuated the induction of IL-1β in addition to the chemokines CXCL2, CXCL11, and LECT1. Interestingly, NRG-1 attenuated the DFP-induced upregulation of paraoxonase-1 (PON1), an enzyme involved in the hydrolysis of organophosphates. The serum concentration of PON-1 is known to be mediated by inflammatory responses [37]. NRG-1 increased brain protein levels of IL-10, an anti-inflammatory cytokine that represses the expression of IL-1, IL-6, and TNFα by activated macrophages [38]. Consistent with this finding, we recently showed that NRG-1 blocked pro-inflammatory responses, induced anti-inflammatory cytokines, and increased survival in a mouse model of cerebral malaria [39]. NRG-1 suppressed the levels of TNFα, IL-6, IL-1α, and CXCL10, while inducing the anti-inflammatory cytokines IL-5 and IL-13. NRG-1 was effective despite having no effect on parasite load during the treatment, suggesting that NRG-1 can alter the immune state to prevent cellular damage following acute CNS injuries.

NRG-1 has previously been shown to affect microglial and macrophage activation [40-46]. NRG-1 reduced free radical release from cultured mouse microglial cells [40], and studies from our laboratory showed that NRG-1 suppressed cyclooxygenase-2 (COX-2) expression in activated human monocyte/macrophage cell cultures [41]. NRG-1 also stimulated microglial proliferation and chemotaxis in a rat model of peripheral nerve injury (PNI) [42,43]. Microglial activation and NRG-1 receptor activation following PNI peaked around 3 days after injury suggesting that microglial activation by NRG-1 may be associated with neuronal repair rather than acute neurotoxicity. The neuropathological and neuroinflammatory sequelae of acute OP poisoning are similar to those observed in other acute CNS injuries, such as stroke, brain trauma, and status epilepticus [15-19,47,48]. Studies from our laboratory and others demonstrated that administration of NRG-1 reduced delayed ischemic brain damage and improved functional recovery in a rat middle cerebral artery occlusion (MCAO) stroke model [32,49-52]. NRG-1 prevented macrophage/microglial activation, reactive astrogliosis, neuronal apoptosis, and pro-inflammatory cytokine expression following stroke [41,50,52]. Taken together, these studies suggest that the neuroprotective efficacy of NRG-1 in DFP-induced brain injury, ischemic stroke, and cerebral malaria might be explained, at least in part, by regulating the immune response and inflammatory mediators.

Recent clinical studies have demonstrated the utility of NRG-1 in human patients with congestive heart failure [53,54]. Phase I clinical studies in China (Chinese Clinical Trial: ChiCTR-TRC-00000414) and Australia (Australian New Zealand Clinical Trials Registry: ACTRN12607000330448) showed that NRG-1 was safe in both healthy and heart failure patients. Recombinant human NRG-1 was used in phase II clinical trials investigating its efficacy in patients with chronic heart failure in both the Australian and Chinese studies [53,54]. In these studies, patients received placebo or NRG-1 at a dose of 0.3 to 1.2 μg/kg/day intravenously for 10 days, in addition to standard drug therapies. During a follow-up period 11 to 90 days after study initiation, NRG-1 significantly improved heart function in patients and the effective doses were shown to be safe and tolerable. Three additional clinical trials to determine the ability of NRG-1 to improve cardiac function after heart failure have been initiated in the US (ClinicalTrails.gov identifiers NCT01258387; NCT01944683; NCT01251406). The doses of NRG-1 that showed efficacy for treating heart failure are near the doses used in our rat stroke and OP studies. In these studies, we showed that NRG-1 was neuroprotective after a single i.a. administration of NRG-1. However, we expect that i.v. administration of NRG-1 will similarly provide neuroprotection as previously demonstrated in other models of acute brain injury [51,55].

In conclusion, our data demonstrate that treatment with NRG-1 blocks the activation of microglia by DFP and decreases DFP-induced pro-inflammatory expression in brain tissues. Our results suggest that NRG-1 protects neurons against DFP-induced delayed cell death by inhibiting toxic pro-inflammatory responses. These findings indicate that NRG-1 has enormous clinical potential and could lead to the development of effective medical countermeasures to facilitate better emergency treatment and protection of civilians and military personnel following exposure to OP nerve agents.
